# Landscape–seascape dynamics in the isthmus between Sørkapp Land and the rest of Spitsbergen: Will a new big Arctic island form?

**DOI:** 10.1007/s13280-014-0572-1

**Published:** 2014-11-11

**Authors:** Wieslaw Ziaja, Krzysztof Ostafin

**Affiliations:** Institute of Geography and Spatial Management, Jagiellonian University, Gronostajowa 7, 30-387 Kraków, Poland

**Keywords:** South Spitsbergen, Climate warming, Glacial recession, Landscape and ecosystem transformation

## Abstract

Transformation of the glaciated isthmus between Sørkapp Land and the rest of Spitsbergen since 1900 is described. The landscape–seascape dynamics depends on the glacial recession determined by climate warming after the Little Ice Age (i.e., since the beginning of the twentieth century, and especially since the 1980s). The isthmus has been narrowed from 28 km in 1899–1900 to 6.2 km in 2013, and lowered by 60–200 m from 1936 to 2005. Two isthmus’ glaciers will have melted, given the current thermic conditions, by 2030–2035. It cannot be ruled out that Sørkapp Land will become an island after that period, because the altitude of the glaciers’ bedrock is close to the sea level. The disappearance of this huge ice mass, even without origin of a sound and island, will lead to a great transformation of the landscape and the ecosystem.

## Introduction and Study area

**Fig. 1:**
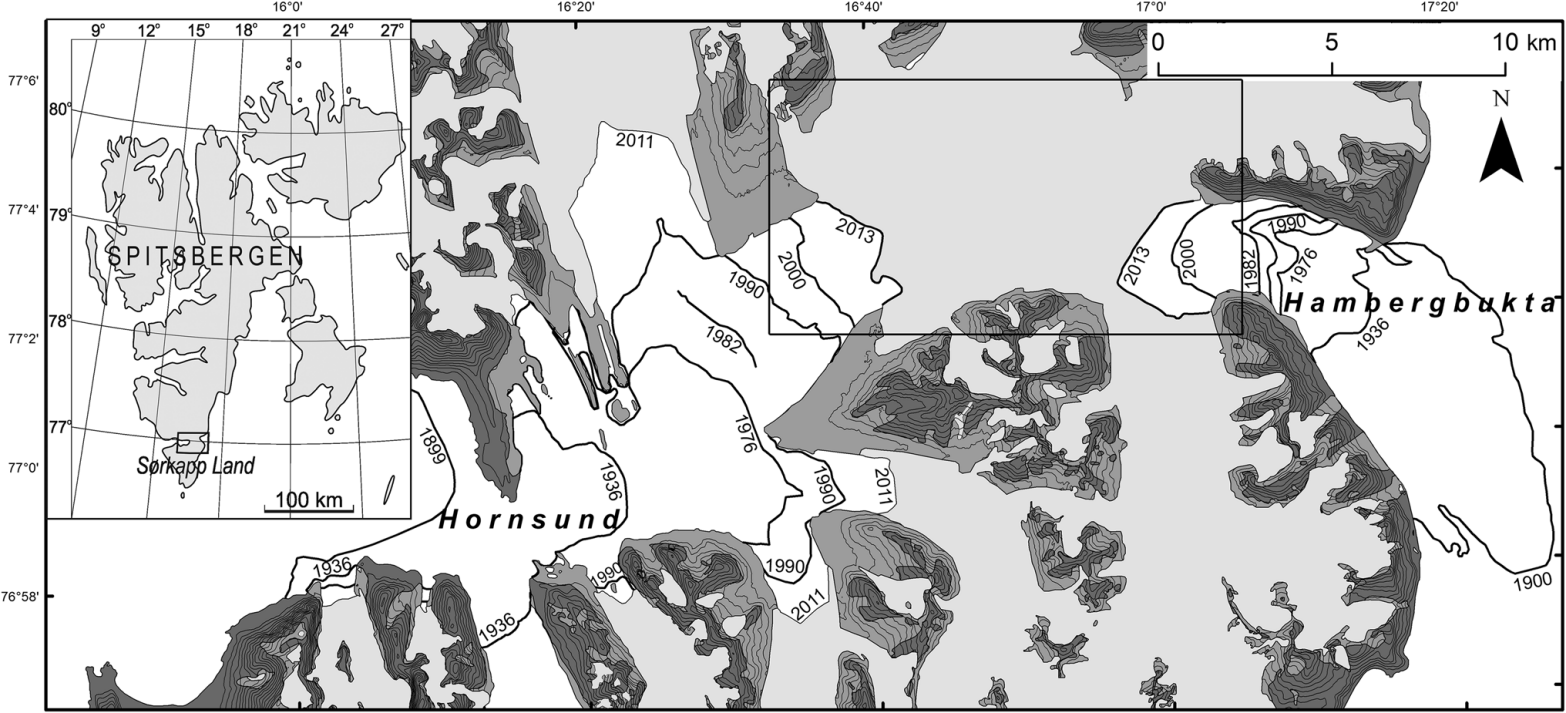
Location and topography of the study area. *White* the sea, *light-gray* land glaciers in 2011; *medium-gray* areas abandoned by glaciers from 1936 to 2011; and *dark-gray* areas free from glaciers in 1936. Frontal extents of the tide-water glaciers are drawn on the basis of: Wassiliew (1925), C12 Markhambreen (1956), C13 Sørkapp (1986), Dallmann et al. (1994), C13 Sørkapp (2007), C12 Markhambreen (2008), Landsat 1–8 and TerraASTER (USGS). Details on the frontal extents are contained in Table 2. The extent of Fig. 2 is marked by the *rectangle*

The study area is located in the Norwegian sector of Arctic at ca. 77°N. The isthmus between Sørkapp Land, the southern Spitsbergen peninsula (ca. 1300 km^2^), and Torell Land is an extraordinarily interesting feature from geographic point of view because it is particularly a dynamic area. It consists of a thick glacial cover settled on bedrock, and it undergoes a quick transformation of its landscape. The isthmus, as the whole Spitsbergen, the biggest island of the Svalbard archipelago, divides the Barents Sea in the east from the Greenland Sea in the west (Fig. 1).

The open Spitsbergen coasts of the aforementioned seas, are in sharp contrast to each other in terms of landscape (Ziaja 2004, 2006, 2012). The climate in the east is much colder than in the west due to the cold East-Spitsbergen Current flowing from the central Arctic to the south, as is evidenced by the extensive glaciation that exists there (Hisdal 1985; Hagen et al. 1993). On the contrary, the western coast is under the influence of the warm West-Spitsbergen Current carrying Atlantic water to the north (Klungsøyr et al. 1995). Such a differentiation, typical for many other Arctic islands, has persisted since the beginning of the Holocene, irrespective of climatic fluctuations.

The Little Ice Age (LIA) ended in Spitsbergen with a cold period during the 1890s (Brázdil 1988). A globally conditioned contemporary warming, with secondary cold and warm fluctuations, began just after 1900 (Førland et al. 1997; Kohler et al. 2002) and still persists today. The current warm and humid fluctuation began in the 1980s (Styszyńska 2005; Walsh et al. 2011; Table 1). The cited quantitative data are from the western coast due to the lack of meteorologic observations in the east.

**Table 1:** Changes in temperature and precipitation at the Polish Polar Station in Hornsund (ca. 30 km west of the isthmus), elaborated on the basis of data from the Institute of Geophysics, Polish Academy of Sciences, published in part by Styszyńska (2013)

Climate features	1980–1989	2000–2009
Mean annual temperature	−5.2°C	−3.15°C
Mean summer, i.e., July and August, temperature	+3.9°C	+4.5°C
Mean annual sum of precipitation	390.5 mm	451.3 mm
Mean summer, i.e., July and August, sum of precipitation	78.1 mm	94.4 mm

**Table 2:** Narrowing of the isthmus between Sørkapp Land and the rest of Spitsbergen for periods ranging from 1899–1900 to 2013

Year	Source	Date of acquisition	The shortest distance between the fronts of Hornbreen and Hambergbreen (m)	Mean annual temperature at the nearest station (°C)^a^
1899–1900	Topographic map 1:200 000 (Wassiliew 1925)	–	28 010	−7.8
1936	Topographic map 1:100 000 (C12 Markhambreen 1956)	–	22 600	−5.7
1976	LANDSAT 1–3 MSS (USGS)	July 17	16 160	−7.8
1982	LANDSAT 4 MSS (USGS)	October 5	–	−5.3
1990	Topographic map 1:100 000 (C12 Markhambreen 2008; C13 Sørkapp 1986), Geological map 1:100 000 (Dallmann et al. 1994)	–	12 238	−2.8
2000	LANDSAT 7 ETM+ (USGS)	June 25	9370	−3.2
2001	Terra ASTER (USGS)	August 17	9055	−3.8
2002	LANDSAT 7 ETM+ (USGS)	June 22	8910	−3.5
2003	Terra ASTER (USGS)	July 24	8229	−5.0
2004	Terra ASTER (USGS)	August 07	8070	−3.6
2005	Terra ASTER (USGS), fieldwork	July 18	7937	−2.6
2006	LANDSAT 7 ETM+ (USGS)	August 14	7349	−1.5
2007	Terra ASTER (USGS)	August 14	7002	−2.4
2009	Terra ASTER (USGS)	July 31	6628	−2.7
2010	LANDSAT 7 ETM+ (USGS)	August 24	6406	−2.5
2011	LANDSAT 7 ETM+ (USGS)	July 28	6295	−2.6
2013	LANDSAT 8 OLI (USGS)	August 24	6167	−2.9

^a^The nearest station: Akseløya for July 1899–June 1900 (data from: Brázdil 1988), Svalbard Airport for 1936 and 1976 (data delivered by the Norwegian Meteorological Institute); and Hornsund for the remaining years (Styszyńska 2013; data delivered by the Polish Polar Station Hornsund)

The isthmus makes a depression between the mountains in the north and south and is also an elevation between the two fjords in the east and west. The isthmus was a part of the extensive ice cover (of the so-called net glaciation) during the LIA, and consisted of two long glaciers being fed by glaciers from the mountains to the south and north of it (Wassiliew 1925; Heinz 1953). Today, after shrinkage or decline of the tributary glaciers from the south, the isthmus resembles a piedmont glacier formed due to coalescence of tributary glaciers from the mountains north of the isthmus. Both parts of the isthmus, Hambergbreen in the east and Hornbreen in the west, are divided by the ice-shed and constitute two fronts of the tide-water glaciers, at the heads of the Hambergbukta and Hornsund fjords (Fig. 1).

Spitsbergen west is built of much older and more resistant rocks than the east; thus, both coasts differ in terrain reliefs from each other. However, the coasts of today’s isthmus are atypical because after the lengthening of the Hornsund fjord (twice during the twentieth century), its head has belonged geologically to Spitsbergen east, being situated 32–33 km east of the open Greenland Sea and only 14–15 km from the open Barents Sea coast. Nowadays, the whole isthmus’ bedrock consists of relatively soft Cretaceous and Tertiary clastic sedimentary rocks (Dallmann et al. 1994), sensitive to erosion and denudation.

Climate changes have always determined the dynamics of feeding the isthmus’ glaciers and thus have shaped the topography of the isthmus. The climate warming has resulted in dramatic environmental and landscape–seascape changes of the isthmus and its surroundings. Since the beginning of the twentieth century, the following processes have occurred permanently: the narrowing and lowering of the isthmus, the lengthening of the Hornsund fjord described recently by Blaszczyk et al. (2013), and the appearance and later lengthening of the Hambergbukta fjord.

These processes are due to global climatic changes influencing local climate, which are partly driven by anthropogenic climate forcing. Locally, however, no direct human disturbance factors are attributable.

The objective of this paper is to show the current landscape–seascape dynamics of the isthmus with its surroundings, and to outline the past and future implications of its reduction.

## Materials and methods

Analyses of the isthmus’ dynamics were made on the basis of examination of the following materials: topographic maps, satellite images, terrain model Aster GDEM, and the results of the authors’ field investigations.

The oldest analyzed topographic map at a scale of 1:200 000 was made by the Russian geodesist, Wassiliew (1925), or Vasiliev, on the grounds of a field survey and observations made during the wintering expedition conducted from 1899 to 1900 (as part of the Russian–Swedish expeditions’ program organized for measurement of the meridian’s length). In spite of inaccuracies in the mountainous areas, this map is a good basis for the analysis of isthmus’ changes as the expedition explored the isthmus many times.

The old topographic map at a scale of 1:100 000, edited by *Norsk Polarinstititutt* (C12 Markhambreen 1956; C13 Sørkapp 1986), was drawn on the basis of oblique air photos taken in 1936. The terrain model with a spatial resolution of 30 m was elaborated on the basis of the vectorized contour lines from this map with an interval of 50 m. The new topographic map at a scale of 1:100 000, edited by the same institute (C13 Sørkapp 2007; C12 Markhambreen 2008), was drawn on the basis of vertical air photos taken in 1961 and 1990. This new map was also scanned and rectified, and its content was vectorized.

Satellite images LANDSAT and TerraASTER (from 1976 to 2013), and the terrain model Aster Global Digital Elevation Model Global Version 2 (actual for 2000–2009) were taken from the collections opened by U.S. Geological Survey (USGS) in www pages http://earthexplorer.usgs.gov/ and http://glovis.usgs.gov/. The spatial resolution of the satellite images is from 60 m (LANDSAT in 1976) to 15 m (TerraASTER). These images are useful for landscape investigations at a scale of 1:100 000 (or a smaller one) and even at a 1:50 000–1:25 000 scale under conditions of scant cloudiness, lack of fog, scant ice cover on the sea, and a lack of snow on the land at the moment of the satellite scanning. A simultaneous occurrence of these conditions is very rare near the isthmus. Hence, only 14 satellite images were selected (from 1976 to 2013), and the glaciers’ extents on them were manually vectorized.

The terrain model was elaborated on for NASA and Japan’s Ministry of Economy, Trade and Industry, which thereafter was released by them on October 17, 2011. This Aster GDEM model was created by stereo-correlating all scenes in the ASTER archive, covering the Earth’s land surface between 83°N and 83°S latitudes. The GDEM is produced with 30-m postings, and is formatted in 1 × 1 degree tiles as GeoTIFF files. The second version of this model was improved after including additional scenes of ASTER. In the ASTER GDEM ver. 2 model, the medium horizontal error is estimated to be less than 10 m, and the medium vertical error may reach up to ca. 15 m (Tachikawa et al. 2011).

**Fig. 2:**
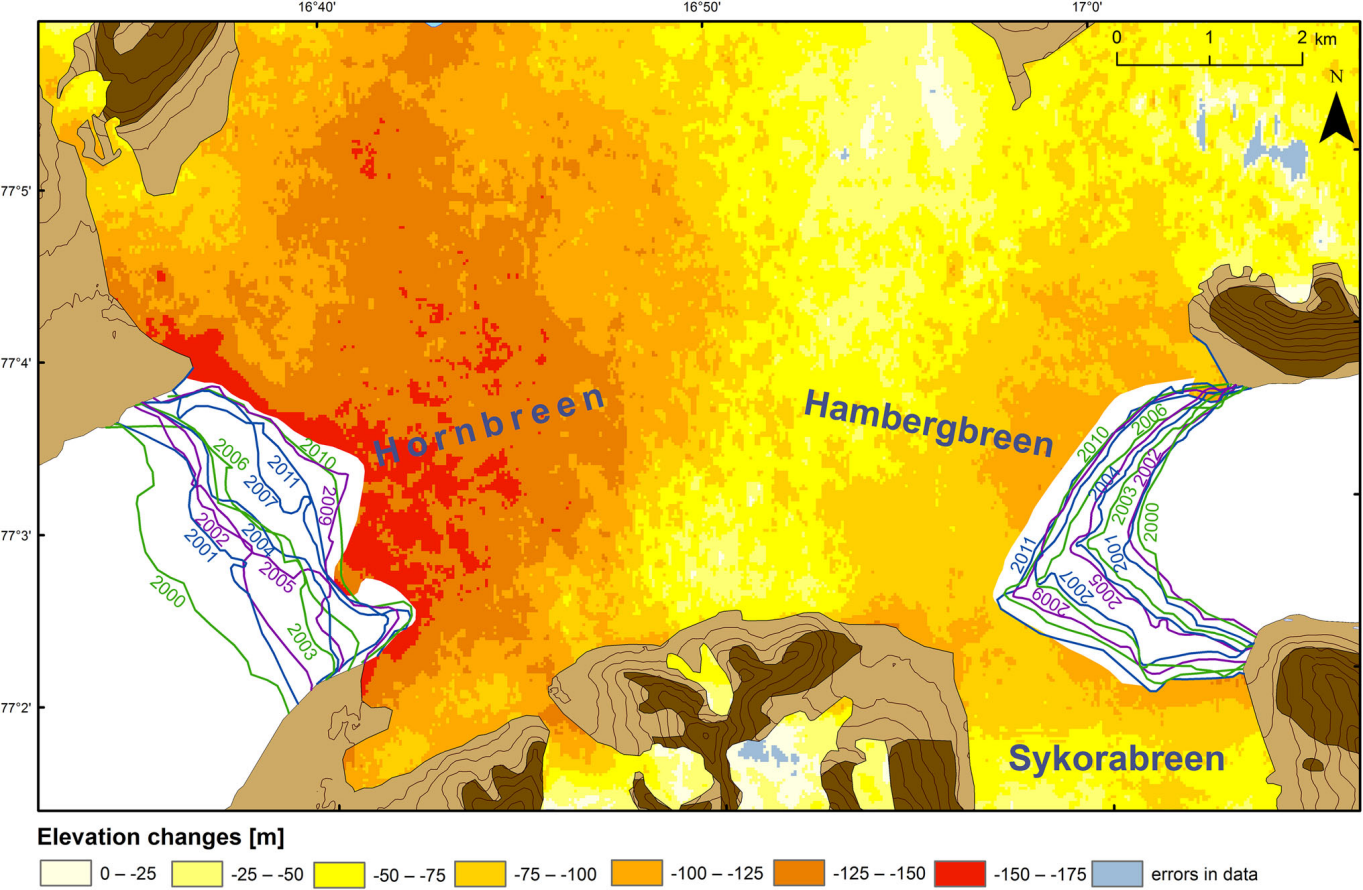
Glaciers’ elevation changes in the today’s (June 24, 2013) isthmus from 1936 to 2000–2009 (on the basis of comparing the raster terrain models: elaborated by the authors for 1936 and taken from USGS for 2000–2009), and annual frontal changes of the isthmus’ tide-water glaciers from 2000 to 2013, apart from 2008 and 2012 (on the basis of USGS)

The map of changes in the glaciers’ elevations and extents (Fig. 2) were elaborated on by comparing the above raster terrain models, using the ArcGIS 10.1 program (*Raster Calculator*).

Our field survey was made using a GPS 12 Garmin and altimeter Paulin. Geographic coordinates and altitudes of many points along profiles and research routes, including extent of glaciers, were measured. The use of these both traditional and modern methods of localization enabled us to minimize mistakes which are specific to each of the methods.

## Results and discussion

The isthmus with its surroundings has undergone a dramatic transformation since the end of the LIA (Figs. 1, 2; and Figs. 4–6 in: Blaszczyk et al. 2009, 2013), due to glacial recession under the climate warming that intensified since the 1980s.

It should be emphasized that the frontal retreat of the isthmus’ ice coasts has been a final result of the negative net mass balance, i.e., a loss of ice mass, of its former and present tributary glaciers (Ziaja 2001; Pälli et al. 2003; Sharov 2006a, b; Blaszczyk et al. 2009). The uplifts of both the equilibrium (of the net mass balance) line altitude (ELA) and the snow line for each glacier have been the direct effect of a (local) rise in temperature, due to the global influence. This caused a lessening of the accumulation zone of each glacier (including its declining and then disappearing, if situated below the new ELA), which has decreased the volume of the ice outflow from this zone. In addition, higher summer temperatures and a longer season without frost have intensified ablation of ice on the glaciers’ surface. As a result, a lowering of the surface elevation and a decrease in the ice thickness have occurred on all the glaciers. This gives rise to the effect of the so-called retreat of the glaciers (which not only is the greatest at their fronts, but also occurs on their sides). Frontal retreat of tide-water glaciers is additionally activated by sea action, intensifying due to a great shortening of the sea–ice season.

**Fig. 3:**
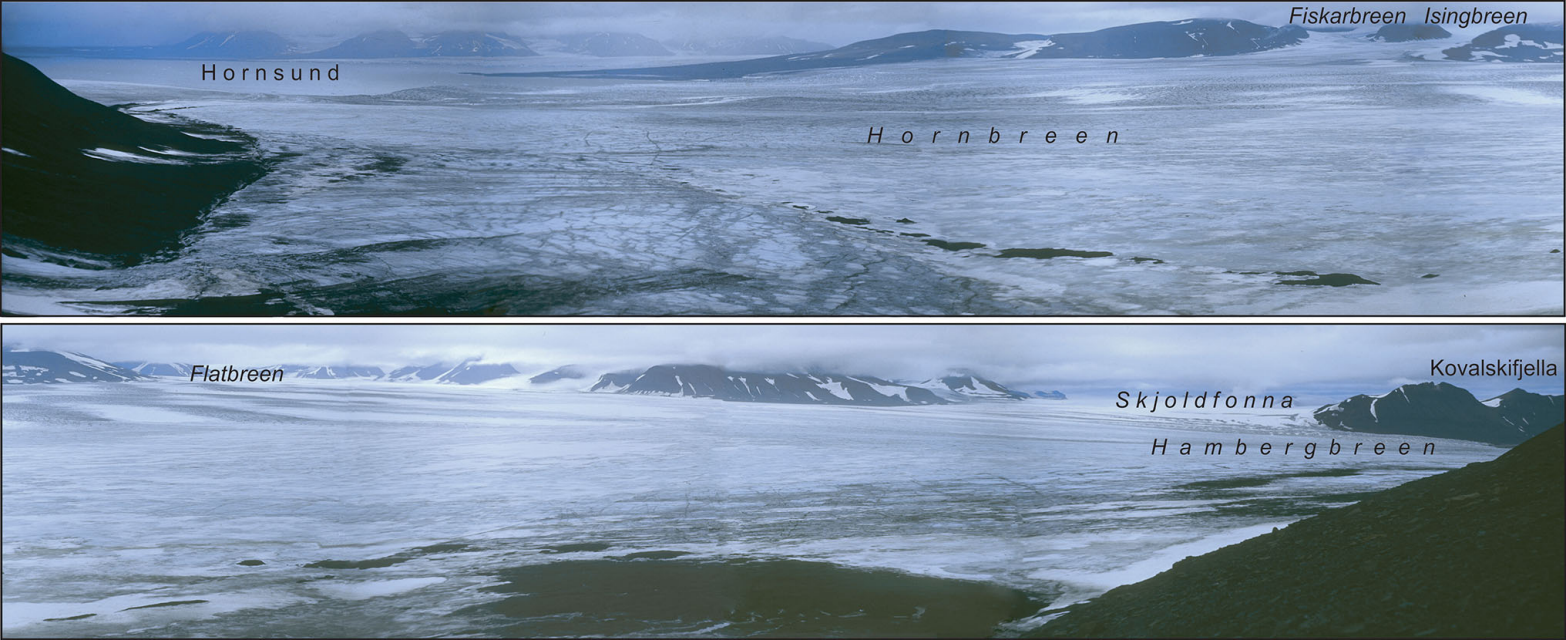
The western (*upper photo*) and eastern (*lower photo*) parts of the isthmus, seen from the south, from ca. 200 m a.s.l., from the slopes of Ostrogradskijfjella, on August 24, 2005. The Hornsund fjord is visible at the left in the background of the upper photo; the frontal part of the Hambergbreen glacier is seen at the right side of the lower photo (the Hambergbukta fjord is not visible). The pass, reaching to 180 m a.s.l., is shown in the foreground of the lower photo. The mountains and glaciers of Torell Land are in the background of both photos (photos by W. Ziaja)

**Fig. 4:**
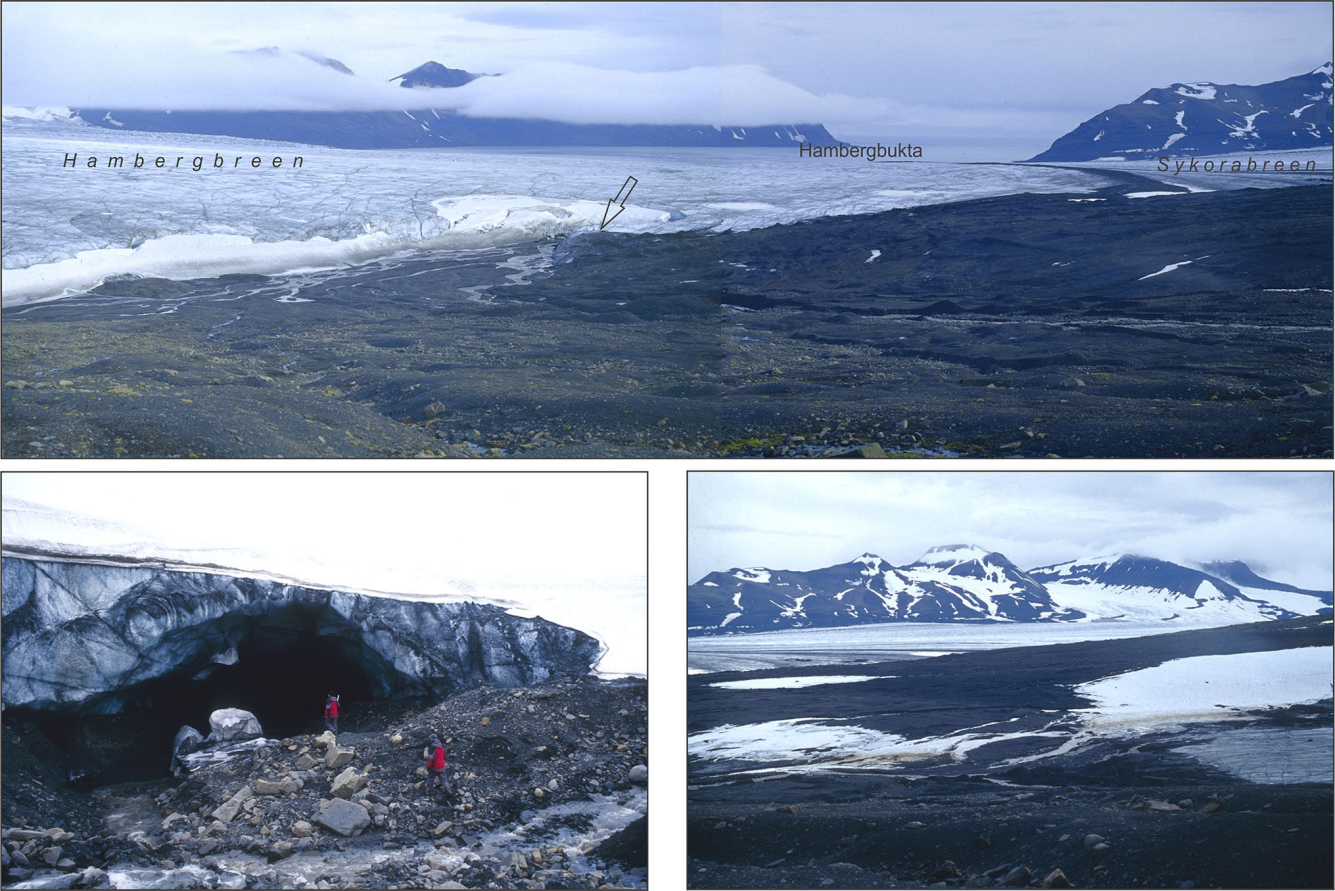
The *upper photo* (from the viewpoint situated at 130–140 m a.s.l. to the east, on August 23, 2005): in the *foreground*, the marginal zone of the Professorbreen glacier, formed after 1970; in the *middle*, the *lowest* *parts* of the Hambergbreen and Sykorabreen glaciers; in the *background*, the Kovalskifjella ridge (*shrouded in fog*) in Torell Land, the Hambergbukta fjord, and the Kamptoppane massif in Sørkapp Land (photo by W. Ziaja). The *lower left photo* (taken by W. Maciejowski, view to the northeast, on 22 August, 2005): tunnel of the subglacial river flowing from the Professorbreen glacier before its recession, preserved at its *inlet* to the Hambergbreen glacier; the tunnel’s location is marked with the arrow in the *upper* *photo*. The *lower right photo* (from the viewpoint at ca. 150–160 m a.s.l. to the southeast, on August 23, 2005): in the *foreground*, the marginal zone and front (near the *right bottom corner*) of the Professorbreen glacier; in the *middle*, the *lower* *part* of the Sykorabreen glacier; in the *background*, the Kamptoppane–Hedgehogfjellet ridge (photo by W. Ziaja)

During 1899–1900, the isthmus was 28 km wide and fed by 21 glaciers (Hornbreen by 15 glaciers and Hambergbreen by 6 glaciers); in 1936, 22.4 km wide and fed by 15 glaciers (Hornbreen by 12 glaciers and Hambergbreen by 3 glaciers); and in 2005, 7.9 km wide and fed by 5 glaciers (Hornbreen by 4 of them and Hambergbreen by only one). Feeding from the south was very insignificant in 2005 because two tributary glaciers’ tongues from this area (Mikaelbreen and the unnamed glacier north of the 584 m peak, which flew to Hornbreen) had narrowed and thinned considerably since the 1980s (Wassiliew 1925; C12 Markhambreen 1956; C13 Sørkapp 1986; Table 2; Figs. 1, 2, 3). The Sykorabreen glacier (Fig. 4), previously the main tributary glacier of Hambergbreen from the south, became the tide-water glacier between 1990 and 1995 (Ziaja 1999).

It is worth noting that the glacial-ice isthmus’ width was longer than the shortest line between the most incised ice cliffs in both glaciers’ heads during the twentieth century (because this line went across the Ostrogradskifjella mountain massif).

Nowadays (2013), the isthmus is only 6.2 km wide and only three glaciers are weakly supplying the isthmus with ice, all of them from the north (Isingbreen and Flatbreen are feeding Hornbreen, and Hambergbreen is being fed by Skjoldfonna). Moreover, the delivery of ice from these glaciers is becoming smaller and smaller due to decreases in their thickness and surface elevation (Fig. 3).

The Hambergbukta fjord did not exist, and its valley was filled with the impressive tongue of the Hambergbreen glacier in 1900. Moreover, its lowest part, comprised a great glacial lobe several km long, protruded into the Barents Sea (Fig. 1).

This protrusion resulted in a huge surge of this glacier just toward the end of the nineteenth century. Afterward, its tongue was shrinking, and its cliff was retreating quickly—by ca. 8 km until 1936, and by 14 km in total until 1957. From 1957 to 1961, the glacier front advanced by 2 km (Lefauconnier and Hagen 1991). We suggest that this may have been the beginning of the next surge. According to Lefauconnier and Hagen (1991), this next surge “likely started in 1961” and persisted until 1970, with a total frontal advance by 5 km in the period between 1961 and 1970. Then, the glacier’s front retreated by 5 km until 1988. From 1988 to 1990, the next smaller surge occurred (Jania 2001), i.e., the lengthening of the glacier by 1.5 km. Subsequently, the glacier was thinning, and its front was retreating quickly, by more than 4 km, until 2013 (Figs. 1, 2). Hence, the ice flux down to the ablation area has been very irregular due to the surges.

Generally, the occurrence of the surges is independent of climatic variation (Lefauconnier and Hagen 1991). However, an increase in the quantity of water at the bottom of a glacier, due to ablation of ice under the warming, may stimulate its surge, which could happen in the study area (Jania 2001). Undoubtedly, a dramatic shrinkage of Hambergbreen and its tributary glaciers has become the main environmental and landscape–seascape implication of the surges there. This happened because the periods of intensified climate warming (or the so-called secondary warm fluctuations) followed just after all the three aforementioned surges. Each time, the higher summer temperature enabled the relatively quick ablation of the huge glacier tongue formed by the surge and hindered rebuilding the accumulation zone (firn field) at the upper parts of the tributary glaciers.

Recession of the western part of the isthmus, i.e., the shrinkage and frontal retreat of the Hornbreen glacier, was much more even and unidirectional during all the periods (since the beginning of the 20th century), in spite of surge episodes mentioned by Pälli et al. (2003). A more regular constant outflow of ice has prevailed in Hornbreen and its tributary glaciers. This may be due to their small inclination (Hambergbreen and its tributary glaciers are significantly steeper). Some exceptions, like the small frontal advance of Hornbreen from 2010 to 2011, did not change this general regularity.

Over a period of ca. 70 years (since 1936), the isthmus’ ice thickness decreased by ca. 60 m in the ice shed between both glaciers and by more below it, down to their fronts. Decrease in the ice thickness reached ca. 125 m near the present (2013) Hambergbreen’s front and 150–175 m near the present (2013) Hornbreen’s front (Fig. 2). Hence, a significantly smaller volume of ice melted on the eastern side of the present isthmus’ area which remains under the influence of the cold East-Spitsbergen Current.

Ice thickness on the isthmus is a basic question in the discussion of the future of both the entire isthmus and its neighborhood in the case of a further glacial recession. Is the bedrock under Hornbreen and Hambergbreen below or above the sea level? If the bedrock is below the sea level, then both fjords, Hornsund and Hambergbukta, will be connected into a sound after the melting of the mentioned glaciers (which is likely in the case of the climate stabilizing or further warming) and Sørkapp Land will be transformed into an island. If not, the isthmus will change its character (from icy to rocky) but Sørkapp Land will remain as the peninsula.

After the discoverer Poole who mistakenly named the fjord “Hornsund”, thinking that it is a sound, in 1610 (The Place-Names of Svalbard 2001), the Russian glaciologist Koryakin (1975) put forward the idea that there is a sub-glacial (buried under ice) sound “dividing Sørkapp Land from the main island of the archipelago” cut in the bedrock of the Hornbreen and Hambergbreen glaciers. He had no direct data on the glaciers’ thicknesses. However, he noticed three indirect circumstances which supported his view: low elevation of the glaciers’ surface, deep sea at the glaciers’ fronts, and a lack of any *nunataks* (isolated rocky peaks which protrude above the glaciers) near the ice-shed.

The next part of this discussion uses the results of radio echolocation surveys of the isthmus’ ice thickness taken from a helicopter. The Norwegian–British team (Drewry et al. 1980) suggested that “Hornbreen is situated on its bedrock in the vicinity, but generally above the sea level and there will be no deep-water connection” by a strait if the glaciers melted. The Russian team (Macheret and Zhuravlev 1985) provided evidence for “the possible existence of a strait under the ice (filled with these two glaciers) separating Sørkapp Land from the rest of (…) Spitsbergen.” It is worth noting that the isthmus’ width was already 12.2 km in 1990 (Table 2).

According to Ziaja (1999, 2001, 2002)—after comparative studies of the papers, topographic maps, air photos, and Landsat satellite images— there appears to be at least a shallow and narrow sound between the Barents Sea and Greenland Sea, and thus the changing of the Sørkapp Land peninsula into an island, is very probable in the event of the glaciers’ melting. He also forecasted the second possibility (if the bedrock would be situated above the sea level): the isthmus being preserved in the form of low (up to a few dozen m) and narrow (up to a few km) land belt (Ziaja 1999).

The “high-resolution ground penetrating radar surveys” (of the isthmus’ ice thickness) at 50 MHz were made by the Finnish–British–Polish team (Pälli et al. 2003) from the surface of the isthmus’ glaciers in April 2000. The surveys’ data, completed with the studies of changes in the glaciers’ surface elevations since 1900 on the topographic maps, led to the following conclusions: (1) Hornbreen and Hambergbreen glaciers are situated below 200 m a.s.l., (2) their beds “lie from –25 to 25 m a.s.l.,” (3) “the low-lying glaciated valley filled by” the glaciers “may become a partially inundated ice-free isthmus within perhaps 100 years,” and (4) “but there is no continuous sub-sea-level channel between Torell Land and Sørkapp Land” (Pälli et al. 2003).

Further studies, based mainly on the satellite interferometry and altimetry (ERS-1/2-SAR interferograms, ICESat-GLAS altimetry data and ASTER-VNIR imagery by 2004), led to the conclusion that “under current environmental conditions” the Hornbreen–Hambergbreen “ice isthmus will disappear by 2020” (Sharov 2006a) and “Sørkapp Land might become a separate island” (Sharov and Osokin 2006).

**Fig. 5:**
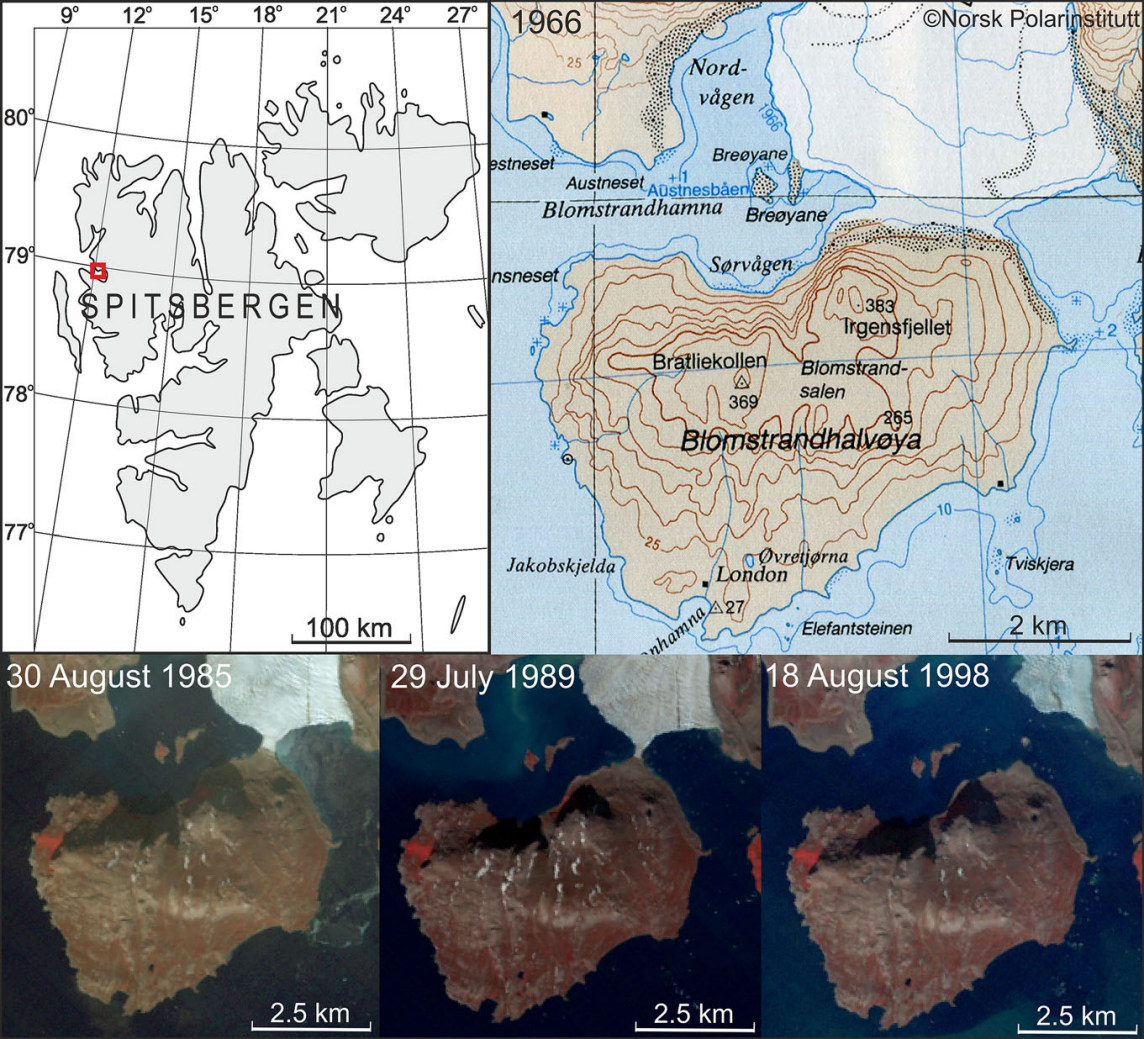
Transformation of the Blomstrandhalvøya peninsula to the new island (A6 Krossfjorden 1987, A7 Kongsfjorden 1990; Landsat satellite data from: USGS for 1985, 1989, 1998)

**Fig. 6:**
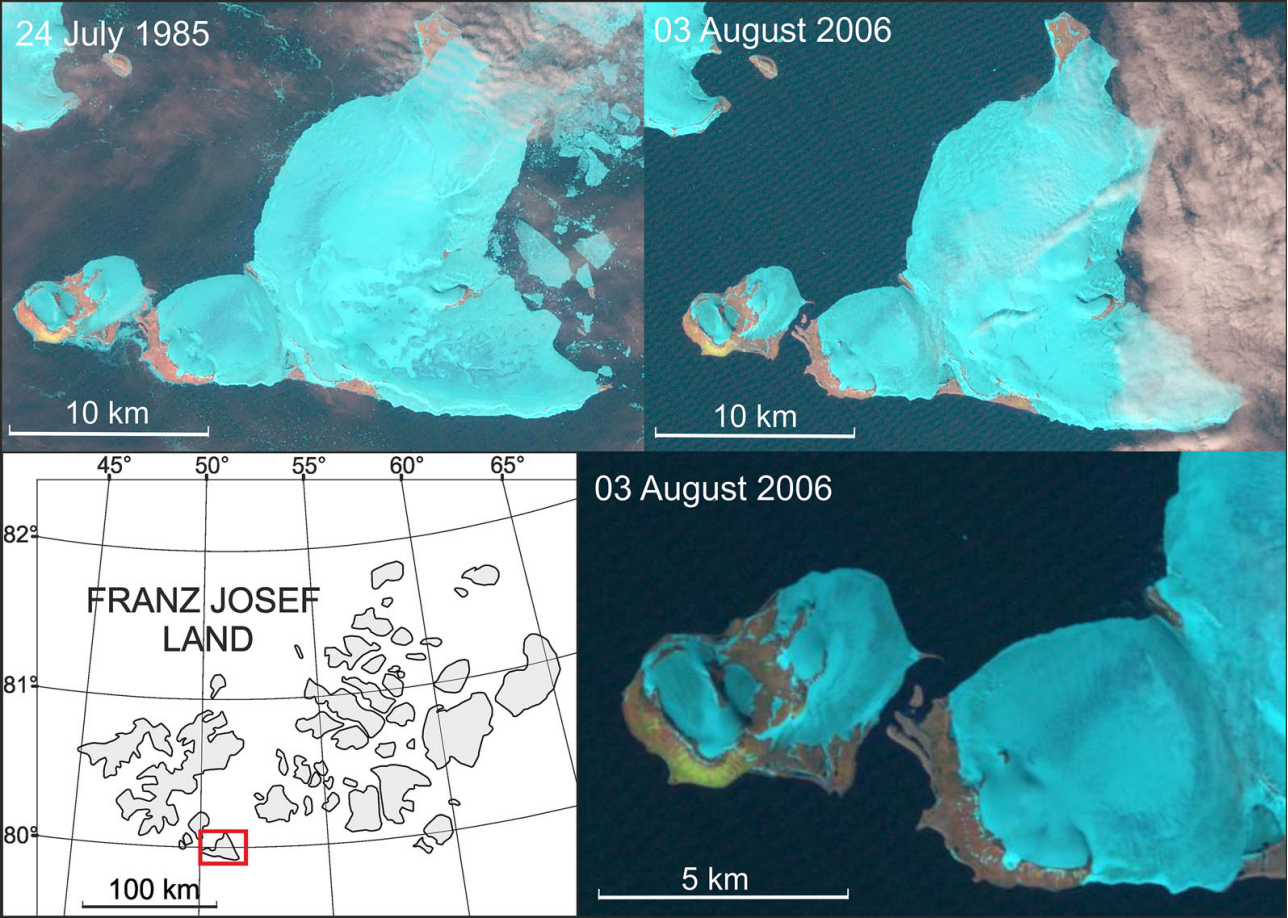
Transformation of the western part of the Northbrook Island to the new Island of Yuriy Kuchiev (Landsat satellite data from: USGS for 1985 and 2006)

Transformation of the Arctic peninsulas to new islands due to the glacier shrinkage is usually described in preliminary or summarizing notes on the Internet or in popular press cited below in this paragraph, being omitted, as it is considered to be an obvious phenomenon, in the main stream of scientific literature on glacial recession. The appearances of new islands in both the eastern Greenland and the western Greenland were noted on the Internet (e.g., World Climate Report 2008; Pelto 2011), whereas glacier recession determining their origin was described in the best glaciological journals (Box and Decker 2011; Howat and Eddy 2011). Such a process was observed in the field by one of the authors of the present study in northwest Spitsbergen where Blomstrandhalvøya was transformed from the peninsula to the new island due to the Blomstrandbreen glacier’s recession by 1995 (Fig. 5). In Franz Josef Land, RussianArctic explorers—first hypothesized that “the new island could have split away from larger Northbrook Island back in 2006” (RIANOVOSTI 2012); discovered the island (Fig. 6) in 2008 (Ostrov Yuriya Kuchieva 2013); and “proved that a new strait has formed” in 2012 (RIANOVOSTI 2012). However, all these islands do not exceed 20 km^2^ and are at least ca. 70 times smaller than Sørkapp Land which would become the biggest new Arctic island in the event of its splitting out from the rest of Spitsbergen.

We made our field survey during a trip on foot across the isthmus: from the Hambergbukta’s south-western coast through the lowest part of the Sykorabreen glacier, and further to the Hornsund fjord along the southern (lowest) isthmus’ edge at the foot of the Ostrogradskijfjella mountainous group, from August 21 to 24, 2005 when the lower parts of the glaciers were devoid of snow (Figs. 1, 2, 3, 4; Ziaja and Ostafin 2005; Ziaja et al. 2007, 2009). Owing to these snowless conditions, the authors of this paper could confirm general regularities discovered by their predecessors, and correct some inaccuracies.

The altitude of the ice pass between Hornsund and Hambergbukta decreased (lowered) from 241 m during 1899–1900 (map 1:200 000: Wassiliew 1925) to ca. 230 m (estimated in the old 1:100 000 map: C12 Markhambreen 1956) in 1936, to 205–210 m in 1961 (estimated in the new 1:100 000 map: C12 Markhambreen 2008), and to ca. 180 m a.s.l. in 2005 (measured by the authors in field). Hence, the mean annual rate of the (glaciers in the) pass lowering has been ca. 0.6 m, similar to the rate ca. 0.5 m reported by Bamber et al. (2005) from “lower elevation glaciers in south Spitsbergen” between 1996 and 2002. The pass also was removed to the south by at least 2 km, just to the foot of the Ostrogradskijfjella’s northern slope (Fig. 3). The thickness of the Sykorabreen glacier (Figs. 1, 2, 4) in the middle of its lower part decreased by ca. 50 m during the period of 1936–2005. This value was obtained by comparing the glacier’s elevation in 1936—red in the old 1:100 000 map (C12 Markhambreen 1956)—with the elevation measured by the authors in the field in 2005.

We also saw that the ELA on Hornbreen and Hambergbreen was significantly underestimated by our predecessors: 250–300 m (Pälli et al. 2003), or 220–250 m (Sharov and Osokin 2006). In fact, the ELAs on all the glaciers explored by us in and near the isthmus (Hornbreen, Hambergbreen, Sykorabreen, Professorbreen, one unnamed glacier, and Mikaelbreen) were surely not lower than 300 m, even in the most shaded northern slopes or valleys of Ostrogradskijfjella. We observed an intensive melting and fissuring of the snowless glaciers below 300 m a.s.l. Moreover, our analysis of the satellite images (LandsatMSS, TerraASTER: USGS) excludes such a significant rise of the ELA during the period of 2000–2005.

On the northern slopes of Ostrogradskijfjella, the traces of a significant lowering (by several dozen m from 1900 to 2005) of the Hornbreen and Hambergbreen glaciers’ surface were very clear (first of all slope incisions and glacial moraines of different types). Contrary to the maps elaborated by Sharov (2006b, c, d), the Professorbreen glacier is completely separated from Hambergbreen due to its shrinkage and shortening. The same refers to the former tributary glaciers of the lower Sykorabreen glacier (Fig. 4).

## Summary and conclusions

Our synthesis of the research results has determined that the future loss of the Hornbreen–Hambergbreen glacial ice isthmus is certain to occur in the current thermic conditions. The low location of the isthmus’ glaciers excludes the possibility of their being rebuilt due to an increase in precipitation. According to Pälli et al. (2003), “Hornbreen and Hambergbreen are unable to build up the reservoir-area mass and geometry for a new surge.”

It can be asked, “What will be the timing of these glaciers’ definite decline?” The time as predicted by Pälli et al. (2003) that “Hornbreen will become ice-free in 82 ± 20 years, and Hambergbreen in 150 ± 50 years” is likely to be too long, and the time as predicted by Sharow (2006a) that “the ice isthmus will disappear by 2020” is likely to be too short.

In our opinion, the ice isthmus will disappear in ca. 20 years, i.e., by the period of 2030–2035 or several years earlier. Our calculation is very simple: the smallest width of the isthmus decreased from 12 328 m in 1990 to 6167 m on June 24, 2013 (Table 2). Hence, the annual rate of its narrowing was ca. 270 m. If the process continues at this rate, then the ice isthmus will disappear in 22–23 years from 2013, i.e., during the period of 2035–2036. However, the process may be accelerated in conjunction with the decrease in the ice volume, which converges with Nuth’s et al.’s (2007, 2010) thesis that the rate of glaciers’ thinning has increased dramatically in south Spitsbergen since 1990. Our projection of the ice isthmus’ disappearance is generally compatible with temperature fluctuations in climate change model projections for this part of the Arctic by the end of the twenty-first century (Overland et al. 2011).

The question of whether Sørkapp Land becomes an island after the disappearance of the ice from the isthmus is impossible to answer given the current state of knowledge (as outlined above), as the research results which refer to altitude of the isthmus’ bedrock are contradictory. Nevertheless, a possibility of the appearance of at least a shallow and narrow sound between the Barents Sea and the Greenland Sea (which would be very interesting not only from a scientific point of view) is not excluded yet. It is worth noting that the Koryakin’s (1975) thesis on a relatively deep incision in the isthmus’ bedrock provided evidence of the lengthening (retreating of the ice heads) of the two fjords over the last 39 years (when any islet appeared from under the retreating glaciers).

It is worth noticing that the contemporary isostatic uplift of Sørkapp Land—less than 1 mm year^−1^ during the last 2000–3000 years (Ziaja and Salvigsen 1995)—is too small to disturb the appearance of the island.

Undoubtedly, the disappearance of such a huge ice mass, even without the origin of a sound and island, will lead to a great transformation of the south Spitsbergen landscape and ecosystem. According to the results of the radar survey, in the case of the ice-freed isthmus’ preserves, it will be very low (below 25 m a.s.l.) and “partially inundated” (Pälli et al. 2003), i.e., with bays and lakes. Hence, it would cease to be a barrier both for the circulation of air masses and living organisms. This would implicate a decline of the current climatic contrast and frequent animal and plant migrations between the eastern and western coasts there (leading, for example, to the appearance of a denser tundra in the east and a greater number of polar bears in the west).

Generally, we consider that the occurrence of glacial surges accelerates landscape transformation processes under a climate change. The surges remove huge ice masses from the accumulation to ablation zone. This accelerates their melting, and thus shrinkage of glaciers, in the periods of climate warming (and the ELA’s rising). In contrast, when the climate becomes colder (and the ELA becomes lower) such removed ice masses melt slowly, which increases the extent of glaciers.
